# Complex Conformational Dynamics of the Heart Failure-Associated Pre-miRNA-377 Hairpin Revealed by Single-Molecule Optical Tweezers

**DOI:** 10.3390/ijms22169008

**Published:** 2021-08-20

**Authors:** Anna Wypijewska del Nogal, Vinoth Sundar Rajan, Fredrik Westerlund, L. Marcus Wilhelmsson

**Affiliations:** 1 Division of Chemistry and Biochemistry, Department of Chemistry and Chemical Engineering, Chalmers University of Technology, SE-412 96 Gothenburg, Sweden; wypijewska.del.nogal@chalmers.se (A.W.d.N.); edal@chalmers.se (V.S.R.); 2 Division of Chemical Biology, Department of Biology and Biological Engineering, Chalmers University of Technology, SE-412 96 Gothenburg, Sweden

**Keywords:** single-molecule force spectroscopy, optical tweezers, RNA dynamics, RNA folding heterogeneity, miRNA, pre-miRNA, pre-miRNA-377, heart failure, RNA-ligand interaction

## Abstract

Pre-miRNA-377 is a hairpin-shaped regulatory RNA associated with heart failure. Here, we use single-molecule optical tweezers to unzip pre-miRNA-377 and study its stability and dynamics. We show that magnesium ions have a strong stabilizing effect, and that sodium ions stabilize the hairpin more than potassium ions. The hairpin unfolds in a single step, regardless of buffer composition. Interestingly, hairpin folding occurs either in a single step (type 1) or through the formation of intermediates, in multiple steps (type 2) or gradually (type 3). Type 3 occurs only in the presence of both sodium and magnesium, while type 1 and 2 take place in all buffers, with type 1 being the most prevalent. By reducing the size of the native hairpin loop from fourteen to four nucleotides, we demonstrate that the folding heterogeneity originates from the large size of the hairpin loop. Further, while efficient pre-miRNA-377 binders are lacking, we demonstrate that the recently developed C2 ligand displays bimodal activity: it enhances the mechanical stability of the pre-miRNA-377 hairpin and perturbs its folding. The knowledge regarding pre-miRNA stability and dynamics that we provide is important in understanding its regulatory function and how it can be modulated to achieve a therapeutic effect, e.g., in heart failure treatment.

## 1. Introduction

**Figure 1:**
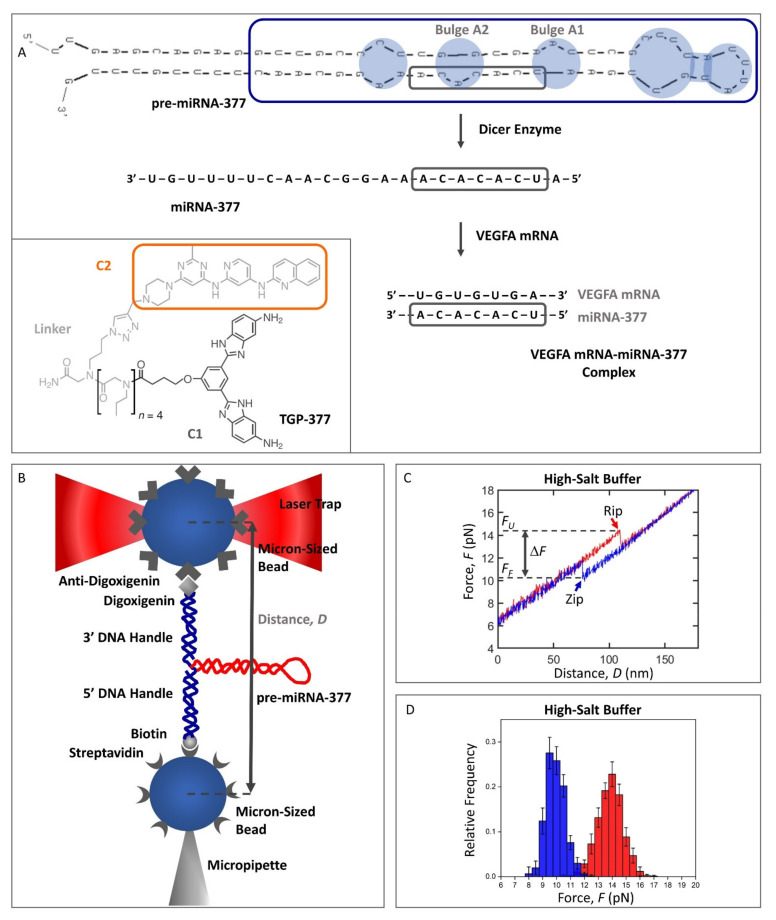
Biosynthesis of miRNA-377 from pre-miRNA-377, which has a role in VEGFA regulation and, hence, in heart failure disease, the experimental setup, and representative force–distance curve (FDC) data from single-molecule optical tweezers folding and unfolding measurements of pre-miRNA-377. (**A**) The single-stranded miRNA-377 is formed from its precursor molecule, the pre-miRNA-377 hairpin, in an enzymatic reaction catalyzed by the Dicer endoribonuclease. It can then form a complex with the VEGFA mRNA via Watson–Crick base-pair complementarity of the depicted sequence fragment (black frame), silencing VEGFA protein expression and suppressing angiogenesis. The blue frame depicts the WT pre-miRNA-377 construct studied herein. The lowest free energy secondary structures of the human full-length pre-miRNA-377 and the construct were predicted in UNAFold. Inset, structure of the dimeric TGP-377 ligand with C1 (dark gray), C2 (framed in orange), and linker (light gray) fragments depicted (adapted from [6]). (**B**) Our optical tweezers setup for studying pre-miRNA-377 stability and dynamics. The pre-miRNA-377 molecule (red) was flanked by a short (29 bp) DNA handle (blue) at each end. The 5′ DNA handle was functionalized with biotin, allowing attachment to a streptavidin-coated bead, which was held still by a micropipette. The 3′ DNA handle was functionalized with digoxigenin, enabling trapping of the other end of the construct with an anti-digoxigenin-coated bead held in a movable and force-measuring laser trap. Drawing is not to scale. (**C**) A representative force–distance curve (FDC) of the WT pre-miRNA-377 unfolding (red) and folding (blue) acquired by optical tweezers. At a median force of 14.1 ± 0.3 pN, the pre-miRNA-377 hairpin unfolds (red arrow—rip; unfolding force—*F_U_*) in a single step, increasing the molecule extension and resulting in a force jump. Moving the laser trap in the opposite direction, and thereby applying a decreasing force leads to pre-miRNA-377 hairpin folding (blue arrow—zip; folding force—*F_F_*) at a median force of 10.1 ± 0.3 pN, also in one step. Δ*F* is the difference between *F_U_* and *F_F_*. The FDC was measured in the high-salt buffer. (**D**) Histogram of the unfolding (red) and folding (blue) forces of the WT pre-miRNA-377 in the high-salt buffer.

Precursor microRNAs (pre-miRNAs) are hairpin-shaped precursors of the short (~22 nt), single-stranded microRNAs (miRNAs; Figure 1A), which are a type of cellular non-coding RNAs that play a regulatory role in gene expression. MiRNAs hybridize to certain protein-coding messenger RNAs (mRNAs; Figure 1A), thereby interfering with their translation in a process called RNA interference (RNAi) [1]. The formation of partially complementary mRNA–miRNA complexes (Figure 1A) regulates gene expression on the level of protein synthesis by a repression of mRNA translation [2] or mRNA degradation [3] and, in a healthy human being, provides homeostasis to the protein abundance. However, elevated or depleted miRNA levels may cause pathological conditions such as cancer as well as neurological, autoimmune, and cardiovascular diseases [1].

Recently, miRNA-377 (Figure 1A) has emerged as a key player in heart failure. A significant increase in miRNA-377 expression was observed in human myocardial tissue from patients with heart failure compared to nonfailing control hearts [4]. Overexpression of miRNA-377 was also shown to lead to increased apoptosis in cardiomyocytes [5]. At the same time, transplantation of human CD34^+^ stem cells deficient in miRNA-377 into the mouse ischemic myocardium was shown to promote their regenerative ability through angiogenesis (blood vessel formation) [4]. The upregulation of human miRNA-377 represses the synthesis of vascular endothelial growth factor A (VEGFA; Figure 1A) and, as a consequence, reduces angiogenesis, which has been associated with heart failure [6,7]. Recently, miRNA-377 has also been identified, using mouse models, to function in the diabetic vascular disease associated with the suppression of the TRAIL cytokine [8]. Heart failure is a highly lethal and increasingly prevalent disease worldwide [4,9,10]. Due to structural or functional abnormalities, the heart is unable to pump the blood sufficiently to meet the needs of the body. With an ageing population and persistently high mortality, new treatments, especially for acute heart failure, are highly demanded [9]. 

A very promising perspective proposed in the treatment of diverse cardiovascular diseases is based on using miRNAs as therapeutic targets, as well as diagnostic biomarkers [11]. However, the identification of small-molecule drugs that selectively modulate one specific upregulated miRNA has been challenging [12]. Moreover, targeting miRNAs by chemically modified antisense oligonucleotides (anti-miRNAs or AMOs) has been limited due to low delivery efficiency, poor tissue distribution, and severe side effects [13]. Therefore, a current R&D approach aims at targeting the structurally more complex pre-miRNAs instead of miRNAs, by small-molecule binders, which have better cellular permeability and broader tissue distribution than oligonucleotides [12,13,14]. This strategy has recently been applied to the cardiovascular disease therapeutic area, resulting in the development of the small-molecule ligand TGP-377, able to selectively bind to the A1 and A2 bulges of the pre-miRNA-377 (Figure 1A) through its C1 and C2 subunits, respectively. TGP-377 has a dissociation constant, *K_D_*, of 190 ± 50 nM [6] and impairs the Dicer enzyme-mediated biosynthesis of miRNA-377 from pre-miRNA-377 (Figure 1) by binding to the Dicer processing site through the C1 subunit. The C2 subunit provides selectivity for pre-miRNA-377 over pre-miRNA-421, which can also bind C1. C2 has a significantly higher binding affinity for pre-miRNA-377 (*K_D_* = 3.0 ± 0.3 μM) compared to C1 (*K_D_* = 65 ± 10 μM); hence, it has been selected herein as the small-molecule ligand for the development of the optical tweezer-based pre-miRNA-binding assay (see Figure 1A for C2 structure).

Previous studies have mainly investigated pre-miRNA-377 as a therapeutic target in human disease [6]. However, the hairpin stability and dynamics have not been fully explored yet, neither for pre-miRNA-377 nor for pre-miRNAs in general. In the present study, we employed single-molecule laser optical tweezers to gain such information. This force spectroscopy technique has emerged as a powerful tool for probing the conformational dynamics of nucleic acids due to its high (down to atomic scale) resolution and sensitivity to rare and transient events [15]. Recent advances in this field have provided new information on DNA and RNA, complementing ensemble techniques in exploring topics ranging from structure (e.g., the intermediate and non-native structures) to mechanics (e.g., how structures are formed and the roles of competing pathways), dynamics (e.g., the intra-molecular interactions in folding and misfolding), and function (e.g., the relationship between dynamics and function) [15]. This is particularly important for RNA, whose regulatory and stimulatory role is greatly dependent on conformational changes, which might be both large-scale rearrangements and subtle fluctuations [16,17]. Additionally, because RNA is prone to degradation at high temperatures (especially in the presence of magnesium ions), optical tweezers experiments that can be carried out at physiological temperatures offer a technical advantage over thermal denaturation experiments (e.g., UV melting) in studying the impact of ions on RNA folding.

Several RNA molecules of various sizes and shapes have been studied by optical tweezers, from simple RNA hairpins made of an A-form double helix and a hairpin loop (e.g., P5ab [18] and HIV-1 frameshifting element [19]) to higher-order structures, such as three-helix junctions, possessing two helices that are coaxially stacked (e.g., P5abcΔA [18]). The list also includes ribozymes (e.g., the P5abc domain of the *Tetrahymena thermophila* ribozyme [18]) and riboswitches (e.g., the *add* adenine riboswitch from *Vibrio vulnificus* [20]). Another popular type of RNA studied by optical tweezers is pseudoknots, formed by the base-pairing of nucleotides from the hairpin loop with complementary nucleotides outside that loop (e.g., the SARS coronavirus pseudoknot [21]). However, to the best of our knowledge, our study presented here is the first investigation of a pre-miRNA using this approach. Many pre-miRNAs have a large hairpin loop (e.g., the hairpin loop of pre-miRNA-377 is 14 nt long; Figure 1A), which makes them structurally distinct from most RNA hairpins studied so far by optical tweezers.

Herein, optical tweezers permitted us to investigate how human pre-miRNA-377 unfolds and folds under physiological conditions. Without using high temperatures or denaturing agents, but by applying a force to the ends of a single pre-miRNA-377 molecule, we were able to directly observe its transition from the folded (hairpin) state to the unfolded state and vice versa. Force and molecule end-to-end distance (extension) were measured and compared between different buffers, containing sodium (Na^+^) or potassium (K^+^) ions in the presence or absence of magnesium (Mg^2+^), providing a measure of the pre-miRNA-377′s mechanical stability in various ionic environments. We also investigated the binding interactions between pre-miRNA-377 and C2, which allowed us to pinpoint the impact of the small molecule on the hairpin’s unfolding and folding. With this study, we gained insight into the pre-miRNA-377 structure–dynamics relationship, which will be beneficial for further structure–function investigations as well as small-molecule ligand design in heart failure therapies directed towards the regulation of miRNA-377 levels.

## 2. Results

### 2.1. Design of the Pre-miRNA-377 Construct for Optical Tweezers Studies

Pre-miRNAs are ~70 nt long hairpins consisting of a non-perfect stem and a terminal hairpin loop [22,23]. Our lowest free energy secondary structure prediction of the full-length, 69 nt long, human pre-miRNA-377 (Figure 1A; sequence from RNAcentral, https://rnacentral.org/rna/URS000075AF10/9606; accessed on 25 February 2021) using UNAFold (Integrated DNA Technologies; https://eu.idtdna.com/UNAFold; accessed on 25 February 2021) showed that it adopts a hairpin structure with four unpaired regions: an A:C mismatch, two A-bulges, and a large hairpin loop (Figure 1A, in blue). A previously reported pre-miRNA-377 structure displays an identical secondary structure of the stem (the same A:C mismatch and the two A-bulges), but it has a different organization of the hairpin loop, where a U-bulge and a CG pair are formed [6], instead of the UG and AU pairs in our prediction (Figure 1A). The latter structure was also obtained by us when we used the RNAstructure software (https://rna.urmc.rochester.edu/RNAstructureWeb/Servers/Fold/Fold.html; accessed on 25 February 2021), instead of UNAFold. This comparison suggests that while the stem is well-defined, different base-stacking interactions are possible within the pre-miRNA-377 hairpin loop, leading to alternative conformations. Irrespective of the tool selected for the secondary structure analysis, all unpaired regions of the pre-miRNA-377, as well as the cleavage site of the Dicer enzyme (which processes pre-miRNAs to RNAi-mediating miRNAs; Figure 1A), are located in the vicinity of the hairpin loop, whereas the 5′ and 3′ ends of the molecule form a double-helix (Figure 1A). From a chemical synthesis point of view, long RNA sequences are challenging to produce, and even more so when they fold back on themselves to form a hairpin [24]. Therefore, we truncated the full-length pre-miRNA-377 to a 50 nt construct (termed wild type, WT pre-miRNA-377; Figure 1A, blue frame), reducing the region of the double-helix, but preserving the unpaired regions. To mitigate end-fraying effects, we truncated the molecule at one of its intrinsic GC pairs. The secondary structure prediction of the WT pre-miRNA-377 construct in UNAFold confirmed that shortening of the stem did not alter the lowest free energy conformation of the rest of the molecule. The theoretical melting temperature, *T_m_*, of the WT pre-miRNA-377 obtained using the UNAFold software was 73 °C (in 1.0 M NaCl), indicating high hairpin stability.

For the optical tweezers experiments, we designed the WT pre-miRNA-377 to be flanked at each end by a double-stranded DNA (dsDNA) only 29 bp in length (see Table S1 for sequence). These dsDNAs served as handles to attach the molecule to beads coated with streptavidin and anti-digoxigenin, respectively (Figure 1B). To the best of our knowledge, short DNA handles have never been used before in optical tweezers studies of RNA molecules, but merely in a few DNA-folding studies [25,26,27,28]. The advantage of using short DNA handles over the more common long handles (typically ~500 bp-a few kbp) [18,29,30,31,32,33] is that they improve signal-to-noise and make the force changes approximately linear with extension (Figure 1C), which significantly benefits the data analysis [25].

The identity and conformity of our construct was investigated in initial experiments in high-salt buffer (1.0 M NaCl; see Figure 1C for a representative force–distance curve, FDC). In this buffer, the WT pre-miRNA-377 unfolded and folded in single steps at a median force of 14.1 ± 0.3 pN (*F_U_*) and 10.1 ± 0.3 pN (*F_F_*), respectively (Figure 1C). The distribution of both the unfolding and folding forces is shown in Figure 1D. The extension of the WT pre-miRNA-377 molecule upon force jumps in unfolding (*E_U_* = 20.8 ± 0.3 nm) and folding (*E_F_* = 21.0 ± 0.5 nm) corresponded to 50 ± 3 nt, confirming that the synthetized construct had the anticipated length.

### 2.2. Impact of Monovalent and Divalent Ions on Pre-miRNA-377 Stability

**Figure 2:**
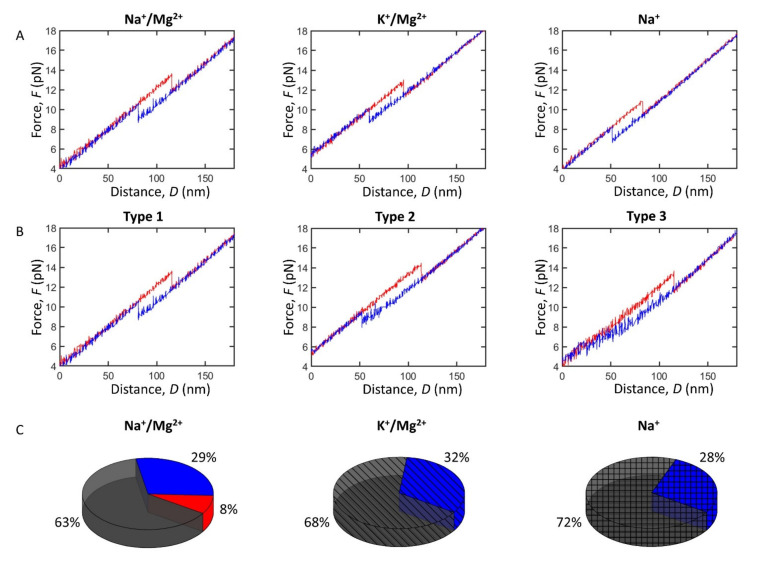
The WT pre-miRNA-377 stability and dynamics. (**A**) Force–distance curves (FDCs) type 1 of the pre-miRNA-377 hairpin’s single-step unfolding (red) and single-step folding (blue) in different buffers. Representative FDCs are plotted, and force distributions can be viewed in Figure S1. (**B**) Three types of pre-miRNA-377 FDCs observed in the Na^+^/Mg^2+^ buffer: type 1 (single-step unfolding and single-step folding), type 2 (single-step unfolding and multi-step folding), and type 3 (single-step unfolding and gradual, i.e., no clear step, folding). Representative FDCs are plotted, and force distributions can be viewed in Figure S1. (**C**) The fractions of folding FDCs for pre-miRNA-377 displaying type 1 (gray), type 2 (blue), and type 3 (red) behavior in different buffers (Na^+^/Mg^2+^—plain, K^+^/Mg^2+^—striped, Na^+^—squared).

The FDCs enabled us to perform a detailed biophysical characterization of the pre-miRNA-377 stability under physiological conditions (Table 1). First, we compared the WT pre-miRNA-377 hairpin unfolding and folding between sodium and potassium buffers (in the presence of magnesium). The major type of FDCs (type 1) obtained in the two buffers is presented in Figure 2A and the corresponding force distribution in Figure S1. We determined that the pre-miRNA-377 hairpin unfolded at a median force of 13.4 ± 0.4 pN in the Na^+^/Mg^2+^ buffer and 12.7 ± 0.4 pN in the K^+^/Mg^2+^ buffer, whereas the folding force was determined to be 8.9 ± 0.2 pN in the Na^+^/Mg^2+^ buffer and 9.0 ± 0.3 pN in the K^+^/Mg^2+^ buffer (Table 1, Figure 2A and Figure S1). Thus, in type 1 FDCs, substituting sodium with potassium ions resulted in a decrease of the unfolding force, while the folding force remained the same (statistical analysis can be found in the Supplementary Materials, Tables S2 and S3). This indicates that potassium ions slightly destabilize the pre-miRNA-377 hairpin compared to the smaller sodium ions.

Next, to examine the impact of divalent metal ions on the pre-miRNA-377 stability, we removed magnesium from the Na^+^/Mg^2+^ buffer. In the Na^+^ buffer, hairpin unfolding occurred at 11.0 ± 0.6 pN and the folding force type 1 was determined to occur at 7.4 ± 0.5 pN (Table 1, Figure 2A and Figure S1). Hence, both unfolding and folding forces decreased significantly in the absence of magnesium (Table 1 and Tables S2 and S3), demonstrating that magnesium has a stabilizing effect on the pre-miRNA-377 hairpin.

### 2.3. Impact of Monovalent and Divalent Ions on Pre-miRNA-377 Dynamics

Apart from gaining an insight into the hairpin stability, single-molecule force manipulations allowed us to monitor structural transitions within pre-miRNA-377 in real time, revealing its unfolding and folding pathways (Figure 2B). We found that, regardless of the buffer composition that we used, unfolding occurred in a single-step rip (Figure 2A,B), where the pre-miRNA-377 hairpin unfolded directly to its fully unfolded state. However, unlike the simple unfolding dynamics, folding FDCs had a more complex pattern and three distinguishable types of curves were found. In addition to a single-step transition as for the unfolding (type 1), FDCs reflecting multi-step folding (type 2) or even a gradual folding without a clear step (type 3) were found (Figure 2B). Type 2 and 3 suggest the formation of intermediates, before the final, full-length hairpin is formed. It is worth noting that the same molecule can show different types of folding behavior, with or without intermediates (see the third column in Table 1, e.g., all eight molecules measured in the K^+^/Mg^2+^ buffer displayed both type 1 and type 2 folding). Furthermore, the unfolding force was independent of folding type (Table 1).

Interestingly, folding behavior type 1 and type 2 were found in all conditions studied, while type 3 folding was observed only in the Na^+^/Mg^2+^ buffer (Figure 2C). Type 1 was dominant in all ionic environments (63–72%; Figure 2C), and type 2 was also significant in all physiological buffers (28–32%; Figure 2C). In the high-salt buffer (1.0 M NaCl), we observed type 2 folding only in approximately 5% of the FDCs. The incidence of type 2 was very similar in all physiological buffers (Figure 2C), suggesting the presence of the multi-step folding process regardless of the counterion used. Type 3, which is specific to the buffer containing both sodium and magnesium ions (Na^+^/Mg^2+^ buffer; Figure 2C), accounted for 8% of all FDCs acquired in this buffer.

Analysis of type 2 FDCs showed that the folding transition occurred at the same median force in the Na^+^/Mg^2+^ (8.1 ± 0.3 pN) and K^+^/Mg^2+^ (8.1 ± 0.4 pN) buffers, and at a significantly lower force in the Na^+^ buffer (7.2 ± 0.4 pN) (Table 1, Figure S1, and Table S3). In conclusion, for both type 1 and type 2 FDCs, magnesium stabilizes the pre-miRNA-377 upon folding, while the replacement of sodium ions with potassium ions does not alter the hairpin folding stability.

The force difference between the unfolding and folding transitions (Δ*F*; Figure 1C) was higher in type 2 and 3 than in type 1 FDCs, regardless of buffer type (Table 1, Figure 2B, Table S4, and Figure S3B), since the folding force was lower. Remarkably, as can be seen in Table 1, the folding extension was much lower in type 2 FDCs (~17 ± 1 nm) compared with type 1 (~20 ± 1 nm), regardless of buffer type, and the distribution of folding extensions was significantly broader (Figure S2). This is because the single-step folding (type 1) is a conformational transition that occurs without the formation of intermediates, while folding by multiple steps (type 2) involves the formation of intermediates. The folding extension in type 3 FDCs was not determined because, in this case, the molecule gradually folds to the hairpin structure.

### 2.4. Loop Mutant of the Pre-miRNA-377

**Figure 3:**
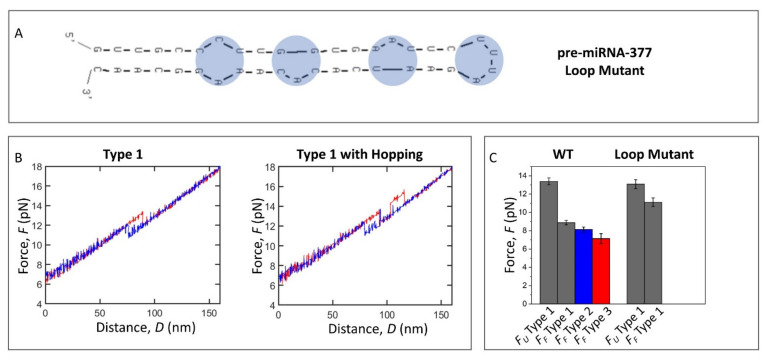
The pre-miRNA-377 loop mutant stability and dynamics. (**A**) The lowest energy secondary structure of the loop mutant predicted in UNAFold. (**B**) Force–distance curves (FDCs) of the loop mutant in Na^+^/Mg^2+^ buffer: type 1 (one single-step unfolding and folding event) and type 1 with hopping (several single-step unfolding and folding events). Representative FDCs are plotted; unfolding—red and folding—blue. Force distribution can be viewed in Figure S4A. (**C**) Unfolding (*F_U_*) and folding (*F_F_*) forces in Na^+^/Mg^2+^ buffer for the WT pre-miRNA-377 and the loop mutant; type 1—gray, type 2—blue, and type 3—red.

To investigate the origin of the complexity of the pre-miRNA-377 conformational dynamics, we designed a loop mutant of the WT pre-miRNA-377. The loop mutant (40 nt; Figure 3A) is identical to the WT pre-miRNA-377 (50 nt; Figure 1A, blue frame) except that it contains a minimal 4 nt UUUA hairpin loop that substitutes the large, native 14 nt hairpin loop of the pre-miRNA-377 (Table S1). Unlike the WT pre-miRNA-377, the lowest free energy secondary structure prediction of the loop mutant (Figure 3A) was identical regardless of the software used (UNAFold or RNAstructure), with an identical stem compared to the WT pre-miRNA-377 (Figure 1A, blue frame vs. Figure 3A), suggesting that changing the hairpin loop does not affect the conformation of the stem region. The lowest free energy, Δ*G*, calculated in UNAFold was −22.4 kcal/mole and −22.2 kcal/mole for the WT pre-miRNA-377 and the loop mutant, respectively.

The FDCs of the loop mutant were obtained in the Na^+^/Mg^2+^ buffer (*N* = 9, *n* = 555) and typical examples are shown in Figure 3B (force histogram is in Figure S4A). Interestingly, we did not observe type 2 or type 3 FDCs for the loop mutant (Figure 3B,C). All transitions between hairpin and unfolded conformations exhibited a single-step character (type 1 FDCs), with a clear rip and a clear zip (Figure 3B). This indicates that the large hairpin loop region of the pre-miRNA-377 is the major cause of the complex folding pathways. Interestingly, several FDCs of the loop mutant showed multiple transitions between the hairpin and the unfolded form (Figure 3B), which occurred both in unfolding (23% of FDCs) and in folding (27% of FDCs). Such multiple transitions in molecule extension between two values within a few piconewtons are termed hopping and indicate bistability [18,34]. Although the loop mutant could hop multiple times during a single pull/relax event, it unfolded and folded completely (type 1 FDCs) at each occasion, and neither type 2 nor type 3 were observed (Figure 3C). This type of bistability has previously been attributed to the free energies of unfolded and folded states being close, and the transition state barrier between the two states being low [34]. Consequently, we suggest that the reduction of the native hairpin loop of the pre-miRNA-377 to a tetraloop lowers the activation energy between the fully unfolded and fully folded states. In turn, since the WT pre-miRNA-377 (Figure 2A,B), unlike the loop mutant (Figure 3B), does not exhibit hopping, but can form intermediates, the large hairpin loop seems to be a structural constraint that causes the pre-miRNA-377 to have more local energy minima in which it can be trapped and kept out of its global thermodynamic equilibrium. In order to quantitatively evaluate the stability of the loop mutant, the force at which the first unfolding/folding event occurred was taken into account for FDCs displaying hopping, similarly to that done before for canonical stem–loop hairpin systems [35]. The unfolding force values were the same for the WT pre-miRNA-377 and the loop mutant (13.4 ± 0.4 pN and 13.1 ± 0.4 pN, respectively; Figure 3C and Table S5), while, in the absence of the extensive loop, the folding force increased to 11.1 ± 0.5 pN, and was significantly higher than for the WT pre-miRNA-377 (8.9 ± 0.2 pN for type 1 FDCs; Figure 3C and Table S5). Accordingly, the loop mutant’s Δ*F* (2.0 ± 0.5 pN) was significantly lower than for the WT pre-miRNA-377 (4.2 ± 0.4 pN for type 1 FDCs; Table S5). As expected, the unfolding and folding extensions of the loop mutant (16.1 ± 0.6 nm and 16.4 ± 0.5 nm, respectively; see Figure S4B for the extension histogram) corresponded to 40 ± 2 nt.

### 2.5. The Effect of C2 Ligand Binding on Pre-miRNA-377 Unfolding and Folding

**Figure 4:**
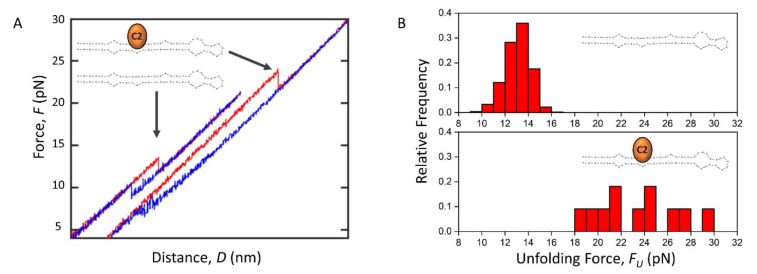
The effect of C2 ligand binding on pre-miRNA-377 unfolding and folding. (**A**) Representative force–distance curves (FDCs) showing the unfolding (red) and folding (blue) of the WT pre-miRNA-377 in absence and presence of 12 μM C2 ligand (orange) in the Na^+^/Mg^2+^ buffer. (**B**) Unfolding force histograms of the WT pre-miRNA-377 without ligand (top) and in presence of 12 μM C2 (bottom) in the Na^+^/Mg^2+^ buffer.

Having characterized the pre-miRNA-377 alone, we investigated the effect of ligand binding on pre-miRNA-377 unfolding and folding. For our experiments, we used the recently developed pre-miRNA-377 ligand: compound 2 (C2, a 4,6-diaminopyrimidine derivative; Figure 1A) [6]. We observed that some unfolding FDCs recorded in the presence of the C2 exhibited a rip at 23.7 ± 1.9 pN (*N* = 5 molecules; Figure 4A,B), which was significantly higher than the unfolding force determined in the absence of the ligand under the same experimental conditions (13.4 ± 0.4 pN; Figure 4A,B). This indicates that C2 stabilizes the pre-miRNA-377 hairpin state. The corresponding folding forces were 7.0 ± 1.6 pN for the ligand-bound state and 8.9 ± 0.2 pN for the most prevalent folding pathway (type 1) in ligand-less conditions, suggesting that the pre-miRNA-377 folds at a significantly lower force in the presence of C2. While 63% of FDCs were type 1 for the ligand-less pre-miRNA-377 (Figure 2C), singe-step folding did not occur at all in the ligand-bound state. Instead, type 3 (gradual folding) and type 2 (multi-step folding) were observed (Figure 4A). Thus, we conclude that the C2 ligand binding to the pre-miRNA-377 stabilizes its hairpin conformation (increases *F_U_*) and, when unfolded, it inhibits folding (decreases *F_F_* and pushes the system completely to type 2 or type 3). The folding inhibition is most likely due to an interaction between the C2 ligand and the unfolded pre-miRNA-377 molecule. Although mechanical enhancement effects have been previously observed in a variety of protein–ligand systems [36] and certain RNA–ligand pairs (e.g., the TERRA G-quadruplex-cPDS small molecule [37] and the *add* adenine riboswitch-adenine [20]), to the best of our knowledge, this is the first case where it has been demonstrated that ligand binding enhances the mechanical stability of a pre-miRNA hairpin and the first pre-miRNA-small-molecule binding assay based on optical tweezers in general.

## 3. Discussion

MiRNAs are powerful gene regulators, whose features and abundance affect human health. Knowledge regarding the stability and conformational dynamics of their precursors, pre-miRNAs, is important for a better understanding of the miRNA biosynthesis and for designing effective therapies stimulating or inhibiting this process, e.g., to treat cancer as well as neurological, autoimmune, and cardiovascular diseases.

Our optical tweezers study provides the first insight into the stability and conformational dynamics of the human pre-miRNA-377, an important therapeutic target in heart failure. These two features are highly dependent on the ionic strength of the solution as well as the counterions used [38,39,40,41]. The size of cations is important for RNA folding [42] and potassium is larger than sodium (Pauling ionic radii are 1.33 Å and 0.95 Å, respectively [43]). Moreover, a potassium ion-dependent structural switch from the canonical stem–loop hairpins to G-quadruplexes has been reported to regulate the Dicer processing of certain pre-miRNAs (e.g., pre-miRNA-92b) [41]. Here, we determined that pre-miRNA-377 adopts a hairpin structure in the presence of both sodium and potassium ions. Our study also showed that the mechanical stability of the hairpin increased slightly (0.7 pN) in the presence of the smaller sodium ions compared to the larger potassium ions (Table 1 and Table S2). Next, we investigated the role of magnesium ions and observed significantly stabilized secondary interactions within the pre-miRNA-377 hairpin in the buffer containing magnesium ions. While the monovalent sodium and potassium ions bind to RNA hairpins diffusely, the divalent magnesium ions display specificity to unpaired regions in the RNA structure, such as bulges and internal loops (Figure 1A), with a *K_D_* in the millimolar range [38]. Since it is expected that small molecules would interact most strongly and specifically with the unpaired regions [44], the binding of a drug might require the displacement of a magnesium counterion. This is particularly interesting in the context of targeting pre-miRNA-377′s bulges adjacent to the Dicer processing site (Figure 1A) with small-molecule ligands in the development of a heart failure therapy.

**Figure 5:**
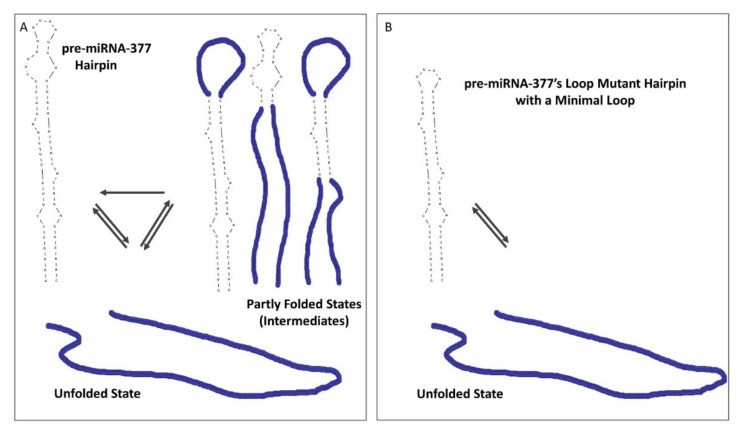
Model of pre-miRNA-377 unfolding and folding. (**A**) The illustrated folded state (hairpin) is the lowest free energy secondary structure of the pre-miRNA-377 predicted in UNAFold. Partly folded states (intermediates) occurring in type 2 and type 3 FDCs are drawn schematically (not predicted). (**B**) With a minimal 4 nt hairpin loop (loop mutant), only transitions without intermediates between the hairpin and the unfolded state are observed. The folded state of the loop mutant illustrates the lowest free energy secondary structure predicted in UNAFold.

By applying optical tweezers, we were also able to disentangle pre-miRNA-377′s folding pathways. It is worth noting that duplex stem–loop hairpins of model DNAs [45,46,47] and RNAs [18] have previously been reported to possess typically an “all-or-none” behavior, where the molecule unfolds and folds in a single step. We found that such a simple two-state conformational dynamics model is not applicable to the pre-miRNA-377 hairpin, and that, instead, folding is a heterogeneous process. In our proposed model (Figure 5), pre-miRNA-377 unfolding is a two-state process, with the initial state being the pre-miRNA-377 hairpin and the final state being its unfolded counterpart. In folding, multiple pathways are possible, where, from the unfolded state, the pre-miRNA-377 may fold to a mature hairpin in a single step (type 1 FDCs) or through partially folded intermediates, which correspond to type 2 and type 3 FDCs (Figure 2B). Regardless of the ionic environment, single-step folding (type 1) is the dominant pathway occurring at ~60–70% of events and multi-step folding (type 2) accounts for ~30% of all FDCs. Folding of type 3, i.e., without a clear step, rarely occurs (~8% FDCs), and it only takes place in the presence of both sodium and magnesium ions. The prevalence of folding pathway type 2, as well as the fact that it is consistently observed in different buffers, indicates that pre-miRNA-377 has the ability to form partially folded hairpins. Importantly, the same molecule can utilize more than one folding pathway in the consecutive unfolding–folding events (Table 1), which highlights the stochastic nature of the folding events.

Interestingly, the folding heterogeneity and the richness of intermediary structures have not been shown before, neither for the pre-miRNA-377 nor for any other pre-miRNA. Future studies will likely show whether these features are conserved among pre-miRNAs and whether they affect gene expression regulation. Recently, the stimulatory function of programmed ribosomal frameshifting (PRF) structures has been attributed to the heterogeneity of their folding dynamics, discriminating the importance of other features, such as unfolding force and rate or barrier height, for stimulation efficiency [21]. Examples range from several pseudoknots (including one from the SARS coronavirus) [21] to the HIV-1 hairpin [19], which all can shift the reading frame of a ribosome on an mRNA in order to generate an alternate gene product. Conformational plasticity has also been suggested to play an important role for an adenine riboswitch [13], which is another type of regulatory RNA structure. Given this literature context, our experimental results call for further investigations into whether the pre-miRNA’s folding heterogeneity and the presence of the pre-miRNA hairpin intermediates serve as a regulatory mechanism in RNAi.

Our investigation also provides a source of understanding of the observed complexity in pre-miRNA-377 dynamics. Reducing the size of the pre-miRNA-377′s hairpin loop to the minimum, four nucleotides, affects its folding pathways. The loop mutant, despite possessing the two bulges and the internal loop of the WT construct, displays an “all-or-none” behavior in unzipping and zipping. Based on these comparative studies of the WT pre-miRNA-377 and its loop mutant, we demonstrate that the folding heterogeneity of the pre-miRNA-377 originates from its large hairpin loop, which results in the formation of local energy minima structures and therefore prevents rapid exchange between fully the folded and fully unfolded states of the molecule.

Finally, we showed for the first time that the optical tweezers technique is powerful for observing the mechanics of ligand binding to pre-miRNA hairpins. Using the previously identified C2 ligand [6], we attributed its efficacy to the mechanical stability enhancement in the pre-miRNA-377 hairpin conformation (C2 shifts the mechanical stability of the pre-miRNA-377 hairpin by ~2 fold). Hence, the C2 subunit most likely helps the inhibitory C1 subunit, which has a lower affinity than C2 (*K_D_* = 65 ± 10 μM vs. 3.0 ± 0.3 μM, respectively [6]), to properly accommodate into the A1 bulge (i.e., the C1-binding site). This is in line with the fact that the *K_D_* of the TGP-377 dimer (190 ± 50 nM [6]) is two orders of magnitude lower than the *K_D_* of C1, indicating a synergistic pre-miRNA-377 binding effect of linking C1 with C2. Furthermore, we found that C2 has the ability to interact with the unfolded pre-miRNA-377, causing an inhibition of its folding. Consequently, we envisage that a new therapeutic approach targeting conformational transitions between the hairpin and the unfolded state may emerge in the treatment of the miRNA-377-associated heart failure and other miRNA-related diseases. Recently, an analogous approach has been successfully demonstrated for mRNAs, where a small molecule called Synucleozid was shown to downregulate the levels of the Parkinson’s disease-related α-synuclein protein by stabilizing the 5′ UTR iron-responsive element (IRE) structure of the α-synuclein-coding mRNA [48].

Apart from increasing the fundamental knowledge of the secondary structure, stability, and dynamics of pre-miRNAs and of the structure–dynamics relationship of RNA hairpins, our study also provides a technical advancement on how to utilize mechanical force ramp and molecular extension to reveal the transient states of pre-miRNAs under physiologically relevant conditions. In addition, for the first time, we demonstrate how to use short dsDNA handles (Table S1) to study RNA by single-molecule optical tweezers to improve the resolution and simplify data analysis.

Studying pre-miRNAs with single-molecule optical tweezers comes with both advantages and disadvantages. Thanks to our single-molecule approach, conformational dynamics could be studied and quantified, allowing us to determine that pre-miRNA-377 forms intermediate structures and that this is due to the large hairpin loop of the molecule. However, further studies are needed to examine the functional consequences of these partially folded structures. In particular, it would be interesting to explore whether they can affect Dicer binding and hence miRNA biosynthesis and/or RNAi-mediated gene expression regulation. Previous studies on other types of regulatory RNAs, such as PRF structures and riboswitches, have shown the role of folding heterogeneity in the regulatory mechanisms of these RNAs [13,19,21]. Therefore, determining the relationship between pre-miRNA-377 folding and regulatory mechanisms will be very interesting and important. Other approaches, especially in cellulo and in vivo techniques, may provide a greater insight into this matter, advancing our understanding of miRNA biology as well as possibly uncovering a new therapeutic strategy to aid in the treatment of heart failure based on the modulation of pre-miRNA folding.

In summary, single-molecule force spectroscopy has enabled us to gain an insight into the structure, stability, and conformational dynamics of a therapeutically very relevant pre-miRNA, the human pre-miRNA-377, which is associated with heart failure. The study highlights the formation of intermediates upon the folding of the pre-miRNA-377 hairpin. This feature of the pre-miRNA could be very interesting for further studies as it may potentially function as a regulatory mechanism in RNAi, by analogy to the folding heterogeneity of other regulatory RNAs reported so far in the literature [13,19,21]. Hence, it could be relevant in the development of therapies targeting pre-miRNAs with small-molecule drugs, aiming at tuning the levels of therapeutically relevant proteins, such as the VEGFA protein (Figure 1A). In addition, we propose that our experimental setup could be used to study mRNA structures and the stabilization of mRNA folds by small-molecule binding, e.g., to halt mRNA translation.

## 4. Materials and Methods

### 4.1. Pre-miRNA-377 Constructs

Two pre-miRNA-377 constructs were studied: WT pre-miRNA-377 (Figure 1A, blue frame) and its loop mutant (Figure 3A). They were purchased as 5′-biotinylated DNA–RNA–DNA hybrid oligonucleotides (see Table S1 for sequences) from Eurogentec, Liège, Belgium (1.0 µmole scale synthesis, PAGE purification, and MS QC). For details on their design, see the Results section.

Lyophilized oligonucleotides were dissolved in RNase-free water to a final concentration of 100 μM. Next, a digoxigenin-labeled tail of ~50 nt was added to the 3′ end of the oligonucleotide. The 3′ modification was performed at 37 °C using Terminal Transferase kit (Merck, Darmstadt, Germany, Ref. #: 3333566001) and at the digoxigenin-11-dUTP (DIG-dUTP; Roche, Rotkreuz, Switzerland, Ref. #: 11558706910) to dATP (Sigma-Aldrich, Deisenhofen, Germany, Ref. #: 11140965001) ratio of 1:10. The reaction was stopped after 15 min by adding 0.5 M EDTA, pH 8.0, yielding 5.0 μM oligonucleotide with the 3′ DIG-dUTP tail. The obtained product was purified at room temperature using the RNeasy Mini Kit (Qiagen, Hilden, Germany, Ref. #: 74104) with 250 mL of ethanol 99.7% for sample preparation and 30 mL of RNase-free water for elution. The purified tailed oligonucleotide was subsequently diluted in RNase-free water to a concentration of 2.0 μM. A 29 nt single-stranded DNA (ssDNA) oligonucleotide complementary to the 5′ and 3′ ssDNA handles (splint) was added to the purified tailed oligonucleotide at an equimolar ratio. Next, they were hybridized in a splint buffer (33 mM Tris-HCl, 167 mM NaCl, and 1.0 mM EDTA; pH 7.4) to obtain the pre-miRNA-377 construct with dsDNA handles, suitable for the optical tweezers’ measurements. The hybridization was carried out in a PCR machine and comprised the following steps: heating at 90 °C for 1.0 min, cooling from 90–80 °C in 10 s, heating at 80 °C for 10 s, cooling from 80–40 °C at 0.5 °C/10 s and from 40–10 °C at 0.5 °C/20 s. The obtained sample was next diluted in RNase-free water to a concentration of 0.2 μM. All the sample preparation steps were carried out in RNase-free PCR tubes. The final sample was aliquoted and stored at −80 °C until its use in the experiments.

The molar absorptivity of the oligonucleotides at 260 nm (1,043,300 M^−1^ cm^−1^ for the WT pre-miRNA-377 and 955,300 M^−1^ cm^−1^ for the loop mutant) was calculated as the sum of molar absorptivities of their 5′ DNA (298,200 M^−1^ cm^−1^), RNA (446,900 M^−1^ cm^−1^ for the WT pre-miRNA-377 and 358,900 M^−1^ cm^−1^ for the loop mutant), and 3′ DNA (298,200 M^−1^ cm^−1^) fragments. For the DNA fragments, the molar absorptivity was approximated by a linear combination of the molar absorptivities of individual nucleotides at 260 nm (*ε*_A_ = 15,300 M^−1^ cm^−1^, *ε*_C_ = 7400 M^−1^ cm^−1^, *ε*_G_ = 11,800 M^−1^ cm^−1^, *ε*_T_ = 9300 M^−1^ cm^−1^ [49]) and multiplied by 0.9 to account for the base-stacking interactions. For the RNA fragment, the molar absorptivity was calculated using OligoAnalyzer (Integrated DNA Technologies; http://eu.idtdna.com/calc/analyzer; accessed on 25 February 2021) and, since the RNA sequence yields a double-helix hairpin, the calculated value was multiplied by 0.9 to account for the base-stacking interactions within the hairpin.

### 4.2. Buffers

Experiments to validate the synthesis of the sample of interest were performed in high-salt buffer (10 mM Tris-HCl, 1000 mM NaCl, and 1.0 mM EDTA; pH 7.4), which yields high resolution in the optical tweezers’ measurements, as a consequence of the high unfolding force required. Experiments to study the impact of divalent metal ions (magnesium ions) were performed in Na^+^/Mg^2+^ buffer (50 mM Tris-HCl, 140 mM NaCl, 5.0 mM KCl, 1.0 mM EDTA, and 5.0 mM MgCl_2_; pH 7.4) vs. Na^+^ buffer (50 mM Tris-HCl, 140 mM NaCl, 5.0 mM KCl, and 1.0 mM EDTA; pH 7.4). Experiments to study the impact of monovalent salt ions (potassium vs. sodium ions) were performed in Na^+^/Mg^2+^ buffer vs. K^+^/Mg^2+^ buffer (50 mM Tris-HCl, 145 mM KCl, 1.0 mM EDTA, and 5.0 mM MgCl_2_; pH 7.4).

### 4.3. Optical Tweezers Measurements of Pre-miRNA-377

Optical tweezers measurements were performed using an in-house built instrument equipped with two counter-propagating 150 mW, 845 nm laser diodes to form a single optical trap. Optical fibers were used to direct the laser beams and approximately 5% of the light was redirected onto a position-sensitive detector (PSD) that recorded the position of the trap. The remaining light was focused through a water-immersion objective lens (60X, NA 1.20) to form the trap. Light exiting the trap was collected by an identical objective lens and directed to a PSD and a photodiode to measure the forces on the trapped beads. Data were recorded at a frequency of 1.0 kHz. The microfluidic chamber was mounted between the objectives and moved using a motorized stage. In an optical tweezers experiment, an anti-digoxigenin-coated bead was captured in the optical trap, while a streptavidin-coated bead was immobilized by suction at the tip of the micropipette (Figure 1B). See Supplementary Materials and Methods for details on the microfluidic chamber and bead preparation. A tether containing a single pre-miRNA-377 construct was established between the two beads and the force–distance measurements were performed by moving the trap at a constant velocity (100 nm/s)—the so-called force–ramp method. Moving the trap away from the micropipette (pull) caused pre-miRNA unfolding, while moving it backwards (relax) resulted in pre-miRNA folding. The movement of the trap changed the distance (*D*; Figure 1B) between the beads and the force (*F*) acting on the hairpin, yielding a force–distance curve (FDC) (Figure 1C). An experiment in a certain buffer was repeated for several hairpin molecules tethered between different pairs of beads (Table 1). Tens to hundreds of FDCs were recorded per single molecule (Table 1). Data are presented as the median ± standard error of the median (SEM), with the size of the sample, *N*, being the number of molecules. The frequency distribution of unfolding and folding forces obtained from multiple acquired FDCs was illustrated in a force histogram (Figure 1D). All measurements were conducted at room temperature (RT; ca. 23 ± 1 °C).

### 4.4. Optical Tweezers Measurements of the C2 Ligand Binding to the Pre-miRNA-377

The small-molecule ligand named compound 2 (C2; Figure 1A), being a subunit of the TGP-377 heterodimer [6], was obtained from AstraZeneca, Gothenburg, Sweden. Experiments with the ligand (12 μM C2) were performed on the WT pre-miRNA-377 in the same conditions as for the ligand-less experiments, i.e., using the force–ramp method at a constant velocity of 100 nm/s, in Na^+^/Mg^2+^ buffer and at RT.

### 4.5. Analysis of Optical Tweezers Data

Folding (relax) events were divided into three different types based on their folding pattern (type 1, 2, or 3) and analyzed separately within each FDC type. The unfolding and folding forces were observed as sudden jumps in FDCs (Figure 1C) and they were extracted using custom-made MATLAB programs. The extension of the pre-miRNA-377 construct (*E*) at a given force (*F*) was obtained using the formula [26]:

(1) $$E\left(F\right)=\frac{\Delta f}{k_{eff}}+x_{d}\left(F\right)$$

where Δ*f* is the measure of a force jump in an FDC, *k_eff_* is the effective stiffness of the hairpin in the folded state (equal to the slope of an FDC), and *x_d_* is the double helix diameter. The *x_d_* was modeled as a single bond of length *d* = 2.0 nm and the mathematical equation for this model is given by:

(2) $$x_{d}\left(F\right)=d\;[coth(\frac{Fd}{k_{B}T})-\frac{k_{B}T}{Fd}]$$

where *k_B_* is the Boltzmann constant and *T* is the absolute temperature (298 K). Force histograms were obtained by combining force values from different FDCs and plotting their relative frequency for the bin size of 0.5 pN. Extension histograms were obtained in an analogous way (bin size of 0.5 nm). The errors reported for each bin in the histograms were obtained by calculating the error of the relative frequency of forces/extensions in each bin over different bead pairs. The force difference (Δ*F*) was determined as the median of individual force differences:

(3) $$\Delta F=Median\;{\left(F_{U}-F_{F}\right)}_{i}$$

where ${\left(F_{U}-F_{F}\right)}_{i}$ is the difference between the unfolding and folding forces of the *i*th FDC (Figure 1C). The pre-miRNA-377 unfolding and folding forces obtained from multiple pull and relax cycles repeated with a given bead pair are dependent on each other, whereas the forces obtained for different bead pairs are independent. Thus, to account for the heterogenous data acquired in the optical tweezers experiments, we used the generalized linear mixed model (GLMM) in our statistical analysis to determine whether there was a significant difference between unfolding forces determined in different buffers and FDC types. The same data treatment was applied for folding forces and force differences. The GLMM test of statistical significance allowed us to consider the random (stochastic) effects present in the experiments. The GLMM analysis was performed in the R software [50,51].

## Figures and Tables

**Table 1:** Force (*F*), extension (*E*), and force difference (Δ*F*) for the WT pre-miRNA-377 unfolding and folding determined by optical tweezers in different buffers ^a^.

Buffer	FDC	*N* ^b^	*n* ^c^	Unfolding		Folding		Δ*F* (pN) ^d^
				*F_U_* (pN)	*E_U_* (nm)	*F_F_* (pN)	*E_F_* (nm)	
High-Salt	Type 1	11/11	765	14.1 ± 0.3	20.8 ± 0.3	10.1 ± 0.3	21.0 ± 0.5	3.9 ± 0.4
	Type 2	8/11	42	-	-	9.2 ± 0.3	18.2 ± 1.3	4.8 ± 0.3
Na^+^/Mg^2+^	Type 1	13/14	545	13.4 ± 0.4	20.9 ± 0.6	8.9 ± 0.2	20.5 ± 0.7	4.2 ± 0.4
	Type 2	13/14	246	-	-	8.1 ± 0.3	16.3 ± 1.0	5.5 ± 0.5
	Type 3	3/14	71	-	-	7.1 ± 0.5	-	5.7 ± 0.9
K^+^/Mg^2+^	Type 1	8/8	453	12.7 ± 0.4	20.2 ± 0.5	9.0 ± 0.3	20.1 ± 0.7	3.5 ± 0.4
	Type 2	8/8	216	-	-	8.1 ± 0.4	16.9 ± 1.1	5.0 ± 0.4
Na^+^	Type 1	5/5	238	11.0 ± 0.6	20.0 ± 0.6	7.4 ± 0.5	19.6 ± 0.9	3.3 ± 0.6
	Type 2	5/5	91	-	-	7.2 ± 0.4	16.7 ± 1.5	4.3 ± 0.4

^a^ Subscripts *U* and *F* refer to unfolding and folding, respectively. The reported values are the median of measurements on three or more molecules ± standard error of the median (SEM). ^b^ Number of molecules showing a given FDC type out of total number of molecules measured in a specific buffer, indicating that the same molecule can utilize more than one folding pathway in consecutive unfolding–folding events. ^c^ Number of FDCs obtained at a given condition. ^d^ Δ*F* was calculated from Equation (3). Measured at room temperature.

## Data Availability

Not applicable.

## Supplementary Materials

The following are available online at www.mdpi.com/article/10.3390/ijms22169008/s1: Supplementary Materials and Methods: Microfluidic chamber and bead preparation, Figure S1: Force histograms of the WT pre-miRNA-377 in different buffers, Figure S2: Extension histograms of the WT pre-miRNA-377 in different buffers, Figure S3: Unfolding and folding forces, and force difference of the WT pre-miRNA-377 in different buffers, Figure S4: Force and extension histograms of the pre-miRNA-377 loop mutant in the Na^+^/Mg^2+^ buffer, Table S1: Oligonucleotide sequences used in optical tweezers experiments, Table S2: Statistical analysis of the WT pre-miRNA-377 unfolding force (*F_U_*) in different buffers, Table S3: Statistical analysis of the WT pre-miRNA-377 folding force (*F_F_*) in different buffers for the three types of folding FDCs, Table S4: Statistical analysis of the WT pre-miRNA-377 force difference (Δ*F*) in different buffers for the three types of folding FDCs, Table S5: Statistical analysis of the WT pre-miRNA-377 vs. loop mutant unfolding force (*F_U_*), folding force (*F_F_*) and force difference (Δ*F*) in the Na^+^/Mg^2+^ buffer for folding FDCs type 1.

## Abbreviations

5′ UTR	5′ untranslated region
Δ*F*	force difference
Δ*G*	free energy
dsDNA	double-stranded DNA
E	extension
E_F_	folding extension
E_U_	unfolding extension
F	force
FDC	force–distance curve
F_F_	folding force
F_U_	unfolding force
IRE	iron-responsive element
K_D_	dissociation constant
miRNA	microRNA
mRNA	messenger RNA
pre-miRNA	pre-microRNA
PRF	programmed ribosomal frameshifting
RNAi	RNA interference
RT	room temperature
ssDNA	single-stranded DNA
T_m_	melting temperature
TRAIL	tumor necrosis factor (TNF)-related apoptosis-inducing ligand
VEGFA	vascular endothelial growth factor A
WT	wild type
