# Supplementary material for: Complex Conformational Dynamics of the Heart Failure-Associated Pre-miRNA-377 Hairpin Revealed by Single-Molecule Optical Tweezers
Source: Int J Mol Sci. 2021 Aug 20;22(16):9008. doi: 10.3390/ijms22169008 (PMC8396532; doi:10.3390/ijms22169008)
Supplement: Supplementary file 1 [file ijms-22-09008-s001.zip › ijms-1290337-supplementary.pdf]

International Journal of *Molecular Sciences*

MDPI

Supplementary Materials

Complex conformational dynamics of the heart failure-associated pre-miRNA-377 hairpin revealed by single-molecule optical tweezers

Anna Wypijewska del Nogal [1,†], Vinoth Sundar Rajan [1,2,†], Fredrik Westerlund [2,*] and L. Marcus Wilhelmsson [1,*]

[1] Division of Chemistry and Biochemistry, Department of Chemistry and Chemical Engineering, Chalmers University of Technology, Gothenburg, SE-412 96, Sweden; wypijewska.del.nogal@chalmers.se (A.W.d.N.); edal@chalmers.se (V.S.R.)
[2] Division of Chemical Biology, Department of Biology and Biological Engineering, Chalmers University of Technology, Gothenburg, SE-412 96, Sweden
* Correspondence: fredrik.westerlund@chalmers.se (F.W.); marcus.wilhelmsson@chalmers.se (L.M.W.)
† These authors contributed equally to this work.

1. Supplementary Materials and Methods

1.1. Microfluidic Chamber and Beads Preparation

The microfluidic chamber had three channels: the upper and lower channels were used to dispense two different types of functionalized beads, while the central channel contained the trap and the micropipette. To remove potential contamination with RNases, the chamber with tubing and syringes were cleaned in a series of subsequent washing steps with RNase-free water, RNase ZAP (Sigma-Aldrich, Deisenhofen, Germany, Ref. #: R2020), RNase-free water, ethanol 99.7%, RNase-free water and finally, with the buffer of interest.

Polystyrene beads coated with streptavidin (Kisker Biotech, Steinfurt, Germany, Ref. #: PC-S-2.0; 2.0 to 2.9 µm diameter) were used to bind the 5′ DNA handle of the pre-miRNA-377 construct that was functionalized with biotin. Anti-digoxigenin beads, to bind the 3′ DNA handle functionalized with digoxigenin, were prepared in-house from Protein G-coated polystyrene beads (Kisker Biotech, Steinfurt, Germany, Ref. #: PC-PG-3.0; 3.0 to 3.4 µm diameter). 1.0 mL of Protein G-coated beads was washed twice with linking buffer (100 mM Na_2HPO_4 and 100 mM NaCl; pH 8.5). The beads, resuspended in the linking buffer, were incubated with 120 µL of anti-digoxigenin polyclonal antibodies (Roche, Rotkreuz, Switzerland, Ref. #: 11333089001) and 60 µL of dimethyl pimelimidate cross-linker (Thermo Fischer Scientific, Waltham, MA, Ref. #: 21666). After incubation, the anti-digoxigenin coated beads were washed twice with PBS buffer (140 mM NaCl, 2.7 mM KCl, 61 mM K_2HPO_4, 39 mM KH_2PO_4, 0.02% NaN_3; pH 7.0) and finally resuspended in 1.0 mL of PBS buffer. The pre-miRNA-377 construct (WT pre-miRNA-377 or Loop Mutant) was incubated with them for 30 min at room temperature, diluted in 1.0 mL of experimental buffer and passed through the upper channel. The streptavidin-coated beads were diluted in experimental buffer and injected into the lower channel.

2. Supplementary Tables

Table S1. Oligonucleotide sequences used in optical tweezers experiments.[a.]

Name	5′-3′ Sequence
miROT1	Biotin-AGTTAGTGGTGGAAACACAGTGCCAGCGC**GUUGCCCUUGGUGAAUUCGCUUU AUUUAUGUUGAAUCACACAAAGGCAAC**AGTTAGTGGTGGAAACACAGTGCCAGCGC
miROT2	Biotin-AGTTAGTGGTGGAAACACAGTGCCAGCGC**GUUGCCCUUGGUGAAUUC UUUAGAAUCACACAAAGGCAAC**AGTTAGTGGTGGAAACACAGTGCCAGCGC

[a] miROT1 and miROT2 contain WT pre-miRNA-377 and Loop Mutant constructs (bold), respectively, flanked by 29 nt long DNA handles at both ends.

Table S2. Statistical analysis of the WT pre-miRNA-377 unfolding force (F_U) in different buffers.[a.]

Buffer-FDC Type	K^+/Mg^{2+} Type 1	Na^+ Type 1
Na^+/Mg^{2+} Type 1	*	***
K^+/Mg^{2+} Type 1	-	***

[a] Statistical analysis was performed in R software using the GLMM statistical test for independent bead pairs and dependent FDCs for a given bead pair. Stars represent the significance factor, *p* values: *** $p \leq 0.001$, ** $p \leq 0.01$, * $p \leq 0.05$ and not significant (NS) $p > 0.05$.

Table S3. Statistical analysis of the WT pre-miRNA-377 folding force (F_F) in different buffers for the three types of folding FDCs.[a.]

Buffer-FDC Type	K^+/Mg^{2+} Type 1	Na^+ Type 1	Na^+/Mg^{2+} Type 2	K^+/Mg^{2+} Type 2	Na^+ Type 2	Na^+/Mg^{2+} Type 3
Na^+/Mg^{2+} Type 1	NS	**	**	-		**
K^+/Mg^{2+} Type 1	-	**	-	*	-	-
Na^+ Type 1	-	-	-	-	NS	-
Na^+/Mg^{2+} Type 2	-	-	-	NS	*	NS
K^+/Mg^{2+} Type 2	-	-	-	-	*	-

[a] Statistical analysis was performed in R software using the GLMM statistical test for independent bead pairs and dependent FDCs for a given bead pair. Stars represent the significance factor, *p* values: *** $p \leq 0.001$, ** $p \leq 0.01$, * $p \leq 0.05$ and not significant (NS) $p > 0.05$.

Table S4. Statistical analysis of the WT pre-miRNA-377 force difference (ΔF) in different buffers for the three types of folding FDCs.[a.]

Buffer-FDC Type	K^+/Mg^{2+} Type 1	Na^+ Type 1	Na^+/Mg^{2+} Type 2	K^+/Mg^{2+} Type 2	Na^+ Type 2	Na^+/Mg^{2+} Type 3
Na^+/Mg^{2+} Type 1	NS	*	***	-	-	*
K^+/Mg^{2+} Type 1	-	NS	-	**	-	-
Na^+ Type 1	-	-	-	-	*	-
Na^+/Mg^{2+} Type 2	-	-	-	NS	**	NS
K^+/Mg^{2+} Type 2	-	-	-	-	NS	-

[a] Statistical analysis was performed in R software using the GLMM statistical test for independent bead pairs and dependent FDCs for a given bead pair. Stars represent the significance factor, *p* values: *** $p \leq 0.001$, ** $p \leq 0.01$, * $p \leq 0.05$ and not significant (NS) $p > 0.05$.

Table S5. Statistical analysis of the WT pre-miRNA-377 *vs*. Loop Mutant unfolding force (F_U), folding force (F_F) and force difference (ΔF) in the Na^+/Mg^{2+} buffer for folding FDCs type 1.[a.]

		pre-miRNA-377 Loop Mutant		
		F_U	F_F	ΔF
WT pre-miRNA-377	F_U	NS	-	-
	F_F	-	***	-
	ΔF	-	-	***

[a] Statistical analysis was performed in R software using the GLMM statistical test for independent bead pairs and dependent FDCs for a given bead pair. Stars represent the significance factor, *p* values: *** $p \leq 0.001$, ** $p \leq 0.01$, * $p \leq 0.05$ and not significant (NS) $p > 0.05$.

3. Supplementary Figures

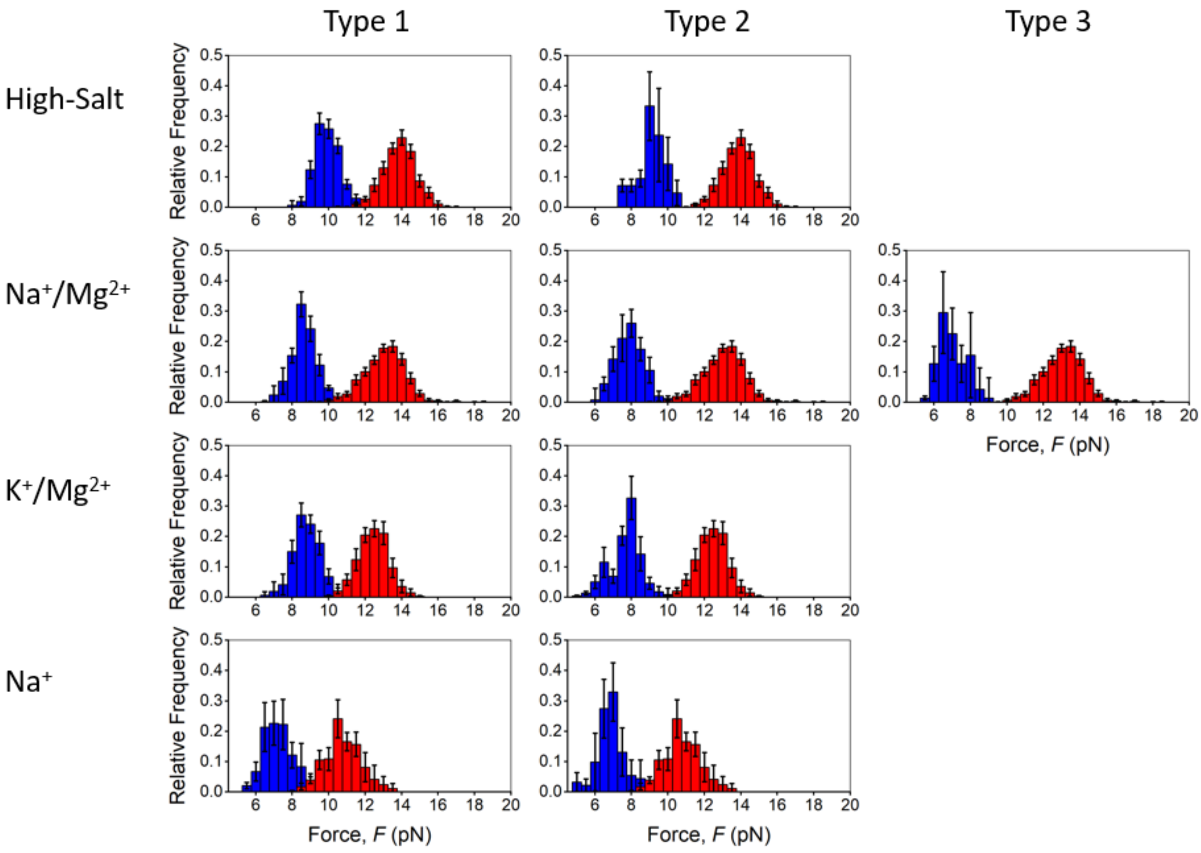

Figure S1. Force histograms of the WT pre-miRNA-377 in different buffers; Unfolding – red and folding – blue.

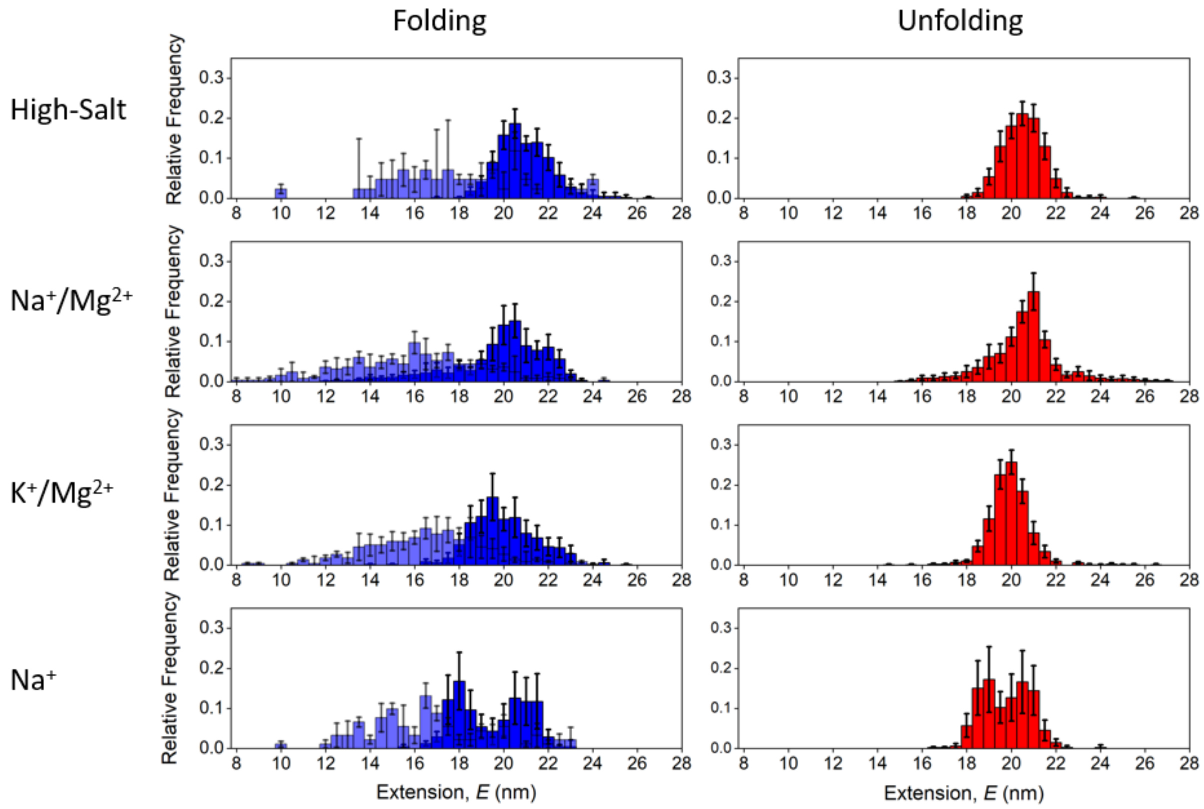

Figure S2. Extension histograms of the WT pre-miRNA-377 in different buffers; Unfolding – red, folding type 1 – blue and folding type 2 – light blue. Folding extension type 3 was not determined because in this case, the molecule adapts the hairpin conformation gradually.

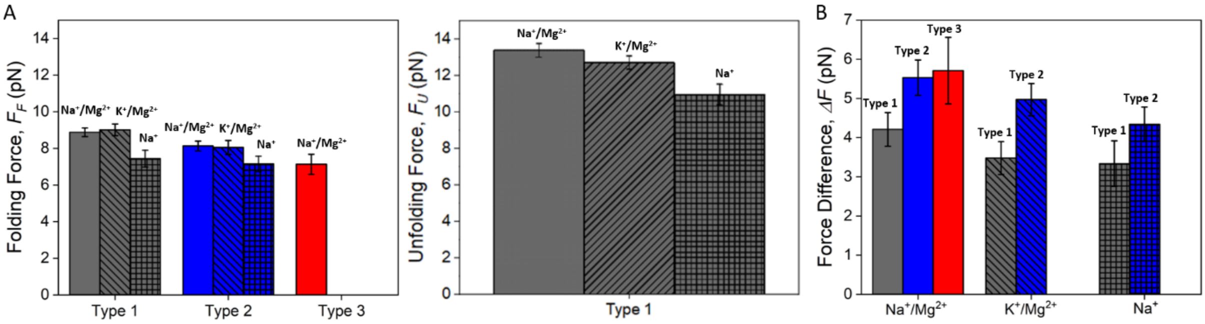

Figure S3. Unfolding and folding forces (**A**), and force difference (**B**) of the WT pre-miRNA-377 in different buffers; Type 1 – gray, type 2 – blue, type 3 – red, Na^+/Mg^{2+} buffer – plain, K^+/Mg^{2+} buffer – striped and Na^+ buffer – squared.

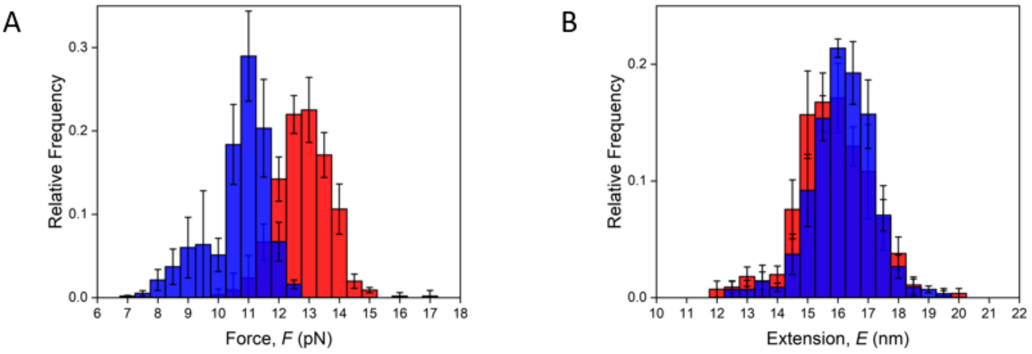

Figure S4. Force (**A**) and extension (**B**) histograms of the pre-miRNA-377 Loop Mutant in the Na^+/Mg^{2+} buffer; Unfolding – red and folding – blue.
